# Effects of aflatoxin and fumonisin on gene expression of growth factors and inflammation-related genes in a human hepatocyte cell line

**DOI:** 10.1093/mutage/geae005

**Published:** 2024-03-12

**Authors:** Hang Wu, Ya Xu, Yun Yun Gong, John Huntriss, Michael N Routledge

**Affiliations:** School of Medicine, University of Leeds, Leeds LS2 9JT, United Kingdom; School of Food Science and Nutrition, University of Leeds, Leeds LS2 9JT, United Kingdom; Guangdong Provincial Key Laboratory of Malignant Tumor Epigenetics and Gene Regulation, Medical Research Center, Sun-Yat University, Guangzhou 51006, China; School of Food Science and Nutrition, University of Leeds, Leeds LS2 9JT, United Kingdom; School of Medicine, University of Leeds, Leeds LS2 9JT, United Kingdom; Leicester Medical School, George Davies Centre, Lancaster Rd, Leicester LE1 7HA, United Kingdom; School of Food and Biological Engineering, Jiangsu University, Zhenjiang 212013, China

**Keywords:** aflatoxin, fumonisin, HHL-16 cells, toxicity, gene expression

## Abstract

Aflatoxin B_1_ (AFB_1_) and fumonisin B_1_ (FB_1_) are mycotoxins widely distributed in maize and maized-based products, often occurring together. The implications of co-exposure to aflatoxin and fumonsin for human health are numerous, but a particular concern is the potential of FB_1_ to modulate AFB_1_ hepatotoxicity. This study evaluated the toxicity of these mycotoxins, alone or combined, in a human non-tumorigenic liver cell line, HHL-16 cells, and assessed the effects of AFB_1_ and FB_1_ on expression of genes involved in immune and growth factor pathways. The results demonstrated that in HHL-16 cells, both AFB_1_ and FB_1_ had dose-dependent and time-dependent toxicity, and the combination of them showed a synergistic toxicity in the cells. Moreover, AFB_1_ caused upregulation of *IL6*, *CCL20*, and *BMP2*, and downregulation of *NDP*. In combination of AFB_1_ with FB_1_, gene expression levels of *IL6* and *BMP2* were significantly higher compared to individual FB_1_ treatment, and had a tendency to be higher than individual AFB_1_ treatment. This study shows that FB_1_ may increase the hepatoxicity of AFB_1_ through increasing the inflammatory response and disrupting cell growth pathways.

## Introduction

Mycotoxins are secondary metabolites of fungi, mainly produced by species from *Aspergillus*, *Fusarium*, and *Penicillium* [1]. Those toxins can contaminate various food crops, posing a threat to human health after consumption of the contaminated foods. Dietary exposure to mycotoxins may cause acute poisoning and chronic health effects, including acute mycotoxicosis, carcinogenicity, immunotoxicity, nephrotoxicity, hepatoxicity, oestrogenic effects, and growth impairment [2].

Aflatoxins that are produced by *Aspergillus flavus* and *A. parasiticus*, contaminate crops such as maize and peanuts in tropical and subtropical areas, because of the climate is favourable fungal growth [3]. In sub-Saharan Africa, maize and peanuts are important staple foods, contributing to a widespread dietary exposure to aflatoxin, particularly in subsistence farming communities [4,5]. Instances of very high exposure to aflatoxin has, from time to time, caused acute aflatoxicosis in Kenya and Tanzania, but generally it is more common to observe the chronic but severe effects of aflatoxin exposure over a longer period [6,7]. In Asia, contamination of rice can also contribute to aflatoxin exposure [8]. Aflatoxin B_1_ (AFB_1_) is the most toxic form of aflatoxin and classified as a group I human carcinogen by the International Agency for Research on Cancer [9]. A high prevalence of hepatocellular carcinoma (HCC) in humans associated with aflatoxin exposure has been observed in Africa and China, especially in populations where hepatitis B virus infection is endemic [10,11]. In addition, the roles of aflatoxin in immune suppression have been reported in various animals and humans [12–14]. Dietary exposure to aflatoxin has also been associated with child growth impairment in several epidemiology studies [15–19]. We have recently reported that dietary aflatoxin exposure is associated with differential DNA methylation in growth factor genes and immune-related genes in Gambian children, which may play a role in aflatoxin-related growth impairment [20,21].

Fumonisin, produced by *Fusarium verticillioides* and *F. proliferatum*, predominantly contaminates maize and maize-based foods in countries with warm climates [22]. Exposure to fumonisin has been associated with liver and oesophageal cancers in populations in Asia and South Africa [23,24], although the evidence is not conclusive and fumonisin is classified as possibly carcinogenic to humans [25]. Fumonisin is non-genotoxic. Due to structural similarity to the long-chain (sphingoid) base backbones of sphingolipids, fumonisin disrupts sphingolipid metabolism, consequently affecting diverse cellular activities, which might contribute to the toxicity and carcinogenicity of fumonisin [26,27]. Risks to neural tube defects in infants were increased for mothers with a large consumption of maize contaminated by fumonisin during the first trimester of pregnancy (odds ratios = 2.4, 95% confidence interval, 1.1–5.3) [28]. The role of fumonisin exposure as a contributor to child growth impairment was reported in Tanzanian children [29,30].

As both aflatoxin and fumonisin carry a number of health risks for humans, the high prevalence of co-occurrence of aflatoxin and fumonisin in foods is of concern, because co-exposure to mycotoxins may have synergistic or additive effects [31,32]. Co-occurrence of aflatoxin and fumonisin was the most observed mixture in approximately 30%, 80%, and 50% of the studies from Africa, Asia, and South America, respectively [33]. Evidence from various animal studies confirms that co-exposure to aflatoxin and fumonisin had synergistic or additive effects on liver damage and inhibition of weight gain [34–36]. In a molecular epidemiology study in Tanzania, a high prevalence of the co-exposure in children suggested that fumonisin exposure alone or together with aflatoxin may contribute to child growth impairment [29,30]. Moreover, it has been posited that aflatoxin and fumonisin exposure may jointly contribute to aetiologies of chronic diseases in humans [37]. One study in the Huaian area of China that quantitatively analysed the biomarkers of aflatoxin and fumonisin in serum and urine samples, found that both aflatoxin and fumonisin exposure levels were significantly higher in cases of oesophageals squamous cell carcinoma (ESCC) than in the control group without ESCC (*P* < 0.05 and 0.01, respectively), with co-exposure to higher levels of aflatoxin and fumonisin being associated with increased risk of ESCC [38]. Such evidence suggests that aflatoxin and fumonsin may at least contribute to child growth impairment and cancer. However, more studies into the molecular mechanism of co-exposure to aflatoxin and fumonisin are required to support this hypothesis.

In this study, we have used an immortalized liver cell line model to investigate the joint effects of aflatoxin and fumonisin. The objectives of this study are to determine: (i) the potential ability of FB_1_ to modulate AFB_1_-induced hepatocytotoxicity; (ii) the effects of single AFB_1_/FB_1_ on gene expression of genes involved in growth factors and immune-related pathways; (iii) the effects of combined AFB_1_ and FB_1_ on gene expression of candidate genes screened from growth factors and immune-related pathways.

## Materials and methods

### Cell lines and chemicals

HHL-16 (human hepatocyte line 16) is a non-tumorigenic human liver cell line derived from primary hepatocytes immortalized by the infection of a retrovirus vector LXSN16E6E7 expressing human papillomavirus type 16 (HPV16) E6 and E7 oncoproteins [39]. The HHL-16 cell line was kindly provided by Dr. Arvind H. Patel (MRC Virology Unit, Glasgow). Cells were cultured in Gibco Minimum Essential Media (MEM) (Thermo Fisher Scientific, UK) supplemented with 10% foetal bovine serum (FBS) and 1% penicillin-streptomycin (Merck Life Science, UK). The cells were maintained at 37 °C with a humidified atmosphere and 5% CO_2_ supplied. The cells were passaged every 2 or 3 days when they reached 80% confluence. Aflatoxin B_1_ (AFB_1_), fumonisin B_1_ (FB_1_), dimethyl sulfoxide (DMSO), and phosphate-buffered saline (PBS) were obtained from Merck Life Science.

### Cell treatments and cell collection

AFB_1_ powder (>98% pure) was dissolved in sterile-filtered DMSO to form a stock concentration of 20 mg/ml, and the final concentrations of AFB_1_ were diluted in a cell culture medium. FB_1_ powder (>98% pure) was dissolved in sterile PBS to give a stock concentration of 10 mg/ml. In single AFB_1_ or FB_1_ treatments, various concentrations of AFB_1_ (1, 5, 10, 20, 50, and 100 µg/ml) and FB_1_ (10, 30, 50, 100, and 150 µg/ml) were used to treat HHL-16 cells for 24 or 48 h. In the combined treatment of AFB_1_ and FB_1_ combinations of 1 µg/ml AFB_1_ + 10 µg/ml FB_1_ (A1_F10), 1 µg/ml AFB_1_ + 50 µg/ml FB_1_ (A1_F50), 1 µg/ml AFB_1_ + 100 µg/ml FB_1_ (A1_F100), 5 µg/ml AFB_1_ + 100 µg/ml FB_1_ (A5_F100), and 10 µg/ml AFB_1_ + 100 µg/ml FB_1_ (A10_F100) were used based on preliminary experiments. After the treatments, the previous media containing AFB_1_ and/or FB_1_ were aspirated and the cells were washed with PBS. The cells were trypsinized for 5 min, new media containing FBS was added to stop the trypsinization, the cells pelleted by centrifugation and the media aspirated out prior to collection of the cell pellet.

### Cell viability assessment

To assess the impact of AFB_1_ and/or FB_1_ on cell viability, the 3-(4.5-dimethylathiazol-2-yl)-2,5-diphenyltetrazolium bromide (MTT) assay was used. HHL-16 cells were seeded into 96-well plates at the density of 2 × 10^4^/well overnight to allow attachment. Various concentrations of single AFB_1_ treatments (see above) in serum-free MEM medium were used to treat cells incubated at 37°C with 5% CO_2_ for 24 and 48 h. DMSO and PBS treatments as control groups for normalization of single AFB_1_ and FB_1_ were used, respectively. After the incubation, 10 µl MTT solution (5mg/ml) (Merck Life Science) was added to each well of cells and incubated for 4 h. Then 100 µl solubilizing solution SDS was added into each well. Finally, the optical density (OD) value was measured at 540 nm and 690 nm using a Multiskan Go Microplate Spectrophotometer (Thermo Fisher Scientific). The result was calculated as a percentage of the control OD value.


$$\begin{array}{l} \%\;of\;cell\;viability=\frac{(A540-A690)\;\;\;sample}{(A540-A690)control}\times 100 \end{array}$$

### Interaction analysis

Combined effects were evaluated by combination index (CI) value determined in the CompuSyn software (ComboSyn Inc., Paramus, NJ, USA) based on the median-effect equation of the mass-action law [40]:


$$\begin{array}{l} log\frac{fa}{fu}=m\times \log(D)-m\times log(Dm), \end{array}$$

where fa and fu represent affected and unaffected fractions, respectively. *D* is the dose of the toxin, *Dm* is the dose required for median effect. *m* is the sigmoidicity coefficient, when *m* > 1 stands for sigmoidal, *m* = 1 hypebolic, and *m* < 1 flat sigmoidal. The linear regression correlation coefficients of the median effect plots were verified and greater than 0.95 [41].

The interaction analysis was conducted by calculating the combination index values for the combination of AFB_1_ and FB_1_ based on the Chou-Talalay method [42]:


$$\begin{array}{l} combination\;\;\;index\;(CI)=\frac{(D)1}{(Dx)1}+\frac{(D)2}{(Dx)2}, \end{array}$$

where (*Dx*)1 and (*Dx*)2 were concentrations of each toxin alone to exert *x*% effect, while (*D*)1 and (*D*)2 represented concentrations of AFB_1_ and FB_1_ in combination to elicit the same effect. CI < 1, =1, and >1 indicates synergistic, additive, and antagonistic effects, respectively.

### RNA extraction, cDNA synthesis, and qPCR

After treatments, HHL-16 cells were collected to extract total RNA using RNeasy Mini Kit (QIAGEN, Manchester, UK) according to the manufacturer’s instructions. Briefly, Buffer RLT was added to homogenize the cells, and add equal volume of 70% ethanol to the homogenized lysate and mixed well by pipetting. The mixture was transferred into an RNeasy spin column and placed in a 2 ml collection tube to centrifuge, followed by Buffer RW1 wash and two-time Buffer RPE washes. Then the RNeasy spin column was placed in a new collection tube and centrifuged to dry the membrane. Finally, RNA was eluted with RNase-free water and the concentration as well as the quality of RNA were determined by a spectrophotometer (DeNovix DS-11).

Next, 1 µg RNA along with TaqMan® Reverse Transcription Kit (Thermo Fisher Scientific) was used to synthesize cDNA under the following condition in a thermal cycler: 25°C/10 min, 37°C/30 min, 95°C/5 min and 4°C/indefinitely.

The mRNA expression levels of interested genes were analysed by qPCR with LightCycler 480 (Roche, UK). Amplifications of the genes were performed in the presence of 5 µl SYBR^TM^ Green PCR Master Mix (Thermo Fisher Scientific) using 4 µl cDNA and 0.5 µl gene-specific primers (Table 1). The primer efficiency of all primers was determined from a standard curve, and the slope of each primer pair was analysed (Table 1). The thermal program for qPCR was 95°C for 10 min, followed by 40 cycles of 95°C for 15 s, 60°C for 1 min, then 95°C for 5 s, 60°C for 1 min, and 95°C/continuous was performed for melting curve analyses to monitor and confirm the accuracy of specific amplification. The cycle threshold (*C*_*q*_) values of all samples were collected and normalized against reference genes beta-actin (ACTB) and glyceraldehyde 3-phosphate dehydrogenase (GAPDH). Relative gene expression was determined using the comparative threshold cycle 2^-ΔΔCT^ method [43].

**Table 1.** qPCR primer sequences.

Gene name	Accession number	Forward (5ʹ to 3ʹ)	Reverse (5ʹ to 3ʹ)	Product size (bp)	Slope of primer efficiency	*R* ^2^
CYP1A2	NM_000761.5	CCTTCGCTACCTGCCTAACC	GTCCCGGACACTGTTCTTGT	124	–	–
CYP3A4	NM_017460.6	CACTCACCCTGATGTCCAGC	TAGGTGGGTGGTGCCTTATTG	75	–	–
CYP3A5	NM_000777.5	TCCTCTATCTATATGGGACCCG	AGCACAGGGAGTTGACCTTC	184	–	–
IL6	NM_000600.5	AGGACATGACAACTCATCTC	GGTGCCCATGCTACATTTGCC	90	−3.6	0.999
CCL20	NM_004591.3	CAAGAGTTTGCTCCTGGCTGC	TTGCTTGCTGCTTCTGATTCGC	75	−3.3	0.999
BMP2	NM_001200.4	CTAAGGAGGACGACAGCACC	AAGAAGTCCCCAGCCAAGTG	109	−3.2	0.996
NDP	NM_000266.4	TCTATGCTCTCCCTGCTGGT	GAGGACAGTGCTGAACGACA	225	−3.5	0.985
ACTB	NM_001101.5	CTGAACCCCAAGGCCAAC	AGCCTGGATAGCAACGTACA	87	−3.5	1
GAPDH	NM_002046.7	GAAGGTGAAGGTCGGAGTCAAC	CAGAGTTAAAAGCAGCCCTGGT	71	−3.5	0.999

### Gene expression analysis of human growth factors pathway

One microgram RNA was used to synthesise cDNA with QuantiTech^®^ Reverse Transcription Kit. One microgram RNA was mixed with 2 µl gDNA Wipeout Buffer, 1 µl internal control RNA, and variable RNase-free water to make up 14 µl in total. After incubating the mixture at 42°C for 2 min, 6 µl Reverse–transcription master mix containing 1 µl Quantiscript Reverse Transcriptase, 4 µl Quantiscript RT Buffer, and 1 µl RT Primer Mix was added into the 14 µl mixture to make a total reaction volume of 20 µl. The reaction was incubated in a thermal cycler as follows: 42°C for 15 min, and 95°C for 3 min.

The synthesized cDNA was used for gene expression analysis of human growth factors genes with the usage of QuantiNova^®^ LNA^®^ PCR Focus Panels (QIAGEN), which contains 84 human growth factors genes (Table 2), six reference genes, three QuantiNova Internal Controls, and three positive PCR controls. Hundred microlitres of the amplified cDNA (20 µl) diluted with RNase-free water (90 µl) was added with 500 µl 2× QuantiNova SYBR Green PCR Master Mix, and 400 µl RNase-free water to make the qPCR components. Ten microlitre of the qPCR components was added to each well of the QuantiNova^®^ LNA^®^ PCR Focus Panels. Roche LightCycler 480 was used for qPCR running with the following thermal program: 95°C for 2 min, followed by 45 cycles of 95°C for 5 s, 60°C for 1 min, then a melting curve was set up as 60°C for 15 s and 95°C/continuous. Cycle threshold (*C*_*q*_) values were uploaded to QIAGEN Web-based data analysis portal (https://geneglobe.qiagen.com/gb/analyze).

**Table 2.** QuantiNova LNA PCR focus panel.

Position	Assay	Name	Symbol	Ensembl ID	Description
A01	SBH1219737	ENST00000221496.4	AMH	ENSG00000104899	anti-Mullerian hormone Source HGNC Symbol Acc HGNC 464
A02	SBH0006040	ENST00000525528.1	BDNF	ENSG00000176697	brain derived neurotrophic factor Source HGNC Symbol Acc HGNC 1033
A03	SBH1219801	ENST00000354870.5	BMP1	ENSG00000168487	bone morphogenetic protein 1 Source HGNC Symbol Acc HGNC 1067
A04	SBH0038117	ENST00000295379.2	BMP10	ENSG00000163217	bone morphogenetic protein 10 Source HGNC Symbol Acc HGNC 20869
A05	SBH1219802	ENST00000378827.5	BMP2	ENSG00000125845	bone morphogenetic protein 2 Source HGNC Symbol Acc HGNC 1069
A06	SBH1219803	ENST00000282701.3	BMP3	ENSG00000152785	bone morphogenetic protein 3 Source HGNC Symbol Acc HGNC 1070
A07	SBH0613995	ENST00000417573.5	BMP4	ENSG00000125378	bone morphogenetic protein 4 Source HGNC Symbol Acc HGNC 1071
A08	SBH1219804	ENST00000370830.4	BMP5	ENSG00000112175	bone morphogenetic protein 5 Source HGNC Symbol Acc HGNC 1072
A09	SBH1219805	ENST00000283147.7	BMP6	ENSG00000153162	bone morphogenetic protein 6 Source HGNC Symbol Acc HGNC 1073
A10	SBH1219806	ENST00000450594.6	BMP7	ENSG00000101144	bone morphogenetic protein 7 Source HGNC Symbol Acc HGNC 1074
A11	SBH0222101	ENST00000372827.8	BMP8B	ENSG00000116985	bone morphogenetic protein 8b Source HGNC Symbol Acc HGNC 1075
A12	SBH0130317	ENST00000399837.8	ADA2	ENSG00000093072	adenosine deaminase 2 Source HGNC Symbol Acc HGNC 1839
B01	SBH0003745	ENST00000221804.5	CLC	ENSG00000105205	Charcot-Leyden crystal galectin Source HGNC Symbol Acc HGNC 2014
B02	SBH1219913	ENST00000420111.6	CSF1	ENSG00000184371	colony stimulating factor 1 Source HGNC Symbol Acc HGNC 2432
B03	SBH1219914	ENST00000296871.4	CSF2	ENSG00000164400	colony stimulating factor 2 Source HGNC Symbol Acc HGNC 2434
B04	SBH0378721	ENST00000225474.6	CSF3	ENSG00000108342	colony stimulating factor 3 Source HGNC Symbol Acc HGNC 2438
B05	SBH0436458	ENST00000610462.1	CSPG5	ENSG00000114646	chondroitin sulfate proteoglycan 5 Source HGNC Symbol Acc HGNC 2467
B06	SBH0404660	ENST00000395761.3	CXCL1	ENSG00000163739	C-X-C motif chemokine ligand 1 Source HGNC Symbol Acc HGNC 4602
B07	SBH0194476	ENST00000373970.4	DKK1	ENSG00000107984	dickkopf WNT signalling pathway inhibitor 1 Source HGNC Symbol Acc HGNC 2891
B08	SBH0331848	ENST00000503921.5	ERAP1	ENSG00000164307	endoplasmic reticulum aminopeptidase 1 Source HGNC Symbol Acc HGNC 18173
B09	SBH0074479	ENST00000244869.3	EREG	ENSG00000124882	epiregulin Source HGNC Symbol Acc HGNC 3443
B10	SBH0534985	ENST00000612258.4	FGF1	ENSG00000113578	fibroblast growth factor 1 Source HGNC Symbol Acc HGNC 3665
B11	SBH0204430	ENST00000575235.5	FGF11	ENSG00000161958	fibroblast growth factor 11 Source HGNC Symbol Acc HGNC 3667
B12	SBH0028571	ENST00000441825.8	FGF13	ENSG00000129682	fibroblast growth factor 13 Source HGNC Symbol Acc HGNC 3670
C01	SBH0177601	ENST00000376143.4	FGF14	ENSG00000102466	fibroblast growth factor 14 Source HGNC Symbol Acc HGNC 3671
C02	SBH0253385	ENST00000518533.5	FGF17	ENSG00000158815	fibroblast growth factor 17 Source HGNC Symbol Acc HGNC 3673
C03	SBH0058115	ENST00000294312.4	FGF19	ENSG00000162344	fibroblast growth factor 19 Source HGNC Symbol Acc HGNC 3675
C04	SBH1220000	ENST00000264498.7	FGF2	ENSG00000138685	fibroblast growth factor 2 Source HGNC Symbol Acc HGNC 3676
C05	SBH0597208	ENST00000215530.6	FGF22	ENSG00000070388	fibroblast growth factor 22 Source HGNC Symbol Acc HGNC 3679
C06	SBH0347299	ENST00000237837.1	FGF23	ENSG00000118972	fibroblast growth factor 23 Source HGNC Symbol Acc HGNC 3680
C07	SBH0225015	ENST00000312465.11	FGF5	ENSG00000138675	fibroblast growth factor 5 Source HGNC Symbol Acc HGNC 3683
C08	SBH0454239	ENST00000228837.2	FGF6	ENSG00000111241	fibroblast growth factor 6 Source HGNC Symbol Acc HGNC 3684
C09	SBH0051743	ENST00000560979.1	FGF7	ENSG00000140285	fibroblast growth factor 7 Source HGNC Symbol Acc HGNC 3685
C10	SBH0087968	ENST00000461657.1	FGF9	ENSG00000102678	fibroblast growth factor 9 Source HGNC Symbol Acc HGNC 3687
C11	SBH1220001	ENST00000297904.4	VEGFD	ENSG00000165197	vascular endothelial growth factor D Source HGNC Symbol Acc HGNC 3708
C12	SBH0309229	ENST00000580279.2	GDF10	ENSG00000266524	growth differentiation factor 10 Source HGNC Symbol Acc HGNC 4215
D01	SBH0256075	ENST00000257868.9	GDF11	ENSG00000135414	growth differentiation factor 11 Source HGNC Symbol Acc HGNC 4216
D02	SBH0310916	ENST00000502572.1	GDNF	ENSG00000168621	glial cell derived neurotrophic factor Source HGNC Symbol Acc HGNC 4232
D03	SBH1220031	ENST00000644934.1	GPI	ENSG00000105220	glucose-6-phosphate isomerase Source HGNC Symbol Acc HGNC 4458
D04	SBH0028682	ENST00000230990.7	HBEGF	ENSG00000113070	heparin binding EGF like growth factor Source HGNC Symbol Acc HGNC 3059
D05	SBH1220091	ENST00000337514.10	IGF1	ENSG00000017427	insulin like growth factor 1 Source HGNC Symbol Acc HGNC 5464
D06	SBH0264962	ENST00000418738.2	IGF2	ENSG00000167244	insulin like growth factor 2 Source HGNC Symbol Acc HGNC 5466
D07	SBH1220095	ENST00000423557.1	IL10	ENSG00000136634	interleukin 10 Source HGNC Symbol Acc HGNC 5962
D08	SBH1220097	ENST00000585513.1	IL11	ENSG00000095752	interleukin 11 Source HGNC Symbol Acc HGNC 5966
D09	SBH1220099	ENST00000231228.2	IL12B	ENSG00000113302	interleukin 12B Source HGNC Symbol Acc HGNC 5970
D10	SBH1220103	ENST00000524595.5	IL18	ENSG00000150782	interleukin 18 Source HGNC Symbol Acc HGNC 5986
D11	SBH0663647	ENST00000263339.3	IL1A	ENSG00000115008	interleukin 1 alpha Source HGNC Symbol Acc HGNC 5991
D12	SBH0079231	ENST00000263341.6	IL1B	ENSG00000125538	interleukin 1 beta Source HGNC Symbol Acc HGNC 5992
E01	SBH0225582	ENST00000226730.4	IL2	ENSG00000109471	interleukin 2 Source HGNC Symbol Acc HGNC 6001
E02	SBH0584080	ENST00000296870.2	IL3	ENSG00000164399	interleukin 3 Source HGNC Symbol Acc HGNC 6011
E03	SBH1220109	ENST00000350025.2	IL4	ENSG00000113520	interleukin 4 Source HGNC Symbol Acc HGNC 6014
E04	SBH1220115	ENST00000243786.3	INHA	ENSG00000123999	inhibin subunit alpha Source HGNC Symbol Acc HGNC 6065
E05	SBH1220116	ENST00000242208.5	INHBA	ENSG00000122641	inhibin subunit beta A Source HGNC Symbol Acc HGNC 6066
E06	SBH1220117	ENST00000295228.4	INHBB	ENSG00000163083	inhibin subunit beta B Source HGNC Symbol Acc HGNC 6067
E07	SBH0407654	ENST00000254958.10	JAG1	ENSG00000101384	jagged 1 Source HGNC Symbol Acc HGNC 6188
E08	SBH0627052	ENST00000546616.1	JAG2	ENSG00000184916	jagged 2 Source HGNC Symbol Acc HGNC 6189
E09	SBH1220168	ENST00000272134.5	LEFTY1	ENSG00000243709	left-right determination factor 1 Source HGNC Symbol Acc HGNC 6552
E10	SBH1221132	ENST00000366820.10	LEFTY2	ENSG00000143768	left-right determination factor 2 Source HGNC Symbol Acc HGNC 3122
E11	SBH1220172	ENST00000249075.4	LIF	ENSG00000128342	LIF, interleukin 6 family cytokine Source HGNC Symbol Acc HGNC 6596
E12	SBH0371009	ENST00000243562.13	LTBP4	ENSG00000090006	latent transforming growth factor beta binding protein 4 Source HGNC Symbol Acc HGNC 6717
F01	SBH0046663	ENST00000395566.8	MDK	ENSG00000110492	midkine Source HGNC Symbol Acc HGNC 6972
F02	SBH0389766	ENST00000260950.4	MSTN	ENSG00000138379	myostatin Source HGNC Symbol Acc HGNC 4223
F03	SBH0209223	ENST00000470584.1	NDP	ENSG00000124479	NDP, norrin cystine knot growth factor Source HGNC Symbol Acc HGNC 7678
F04	SBH0318562	ENST00000369512.2	NGF	ENSG00000134259	nerve growth factor Source HGNC Symbol Acc HGNC 7808
F05	SBH0463463	ENST00000287139.7	NODAL	ENSG00000156574	nodal growth differentiation factor Source HGNC Symbol Acc HGNC 7865
F06	SBH0274670	ENST00000652592.1	NRG1	ENSG00000157168	neuregulin 1 Source HGNC Symbol Acc HGNC 7997
F07	SBH0471786	ENST00000361474.6	NRG2	ENSG00000158458	neuregulin 2 Source HGNC Symbol Acc HGNC 7998
F08	SBH0290718	ENST00000545131.5	NRG3	ENSG00000185737	neuregulin 3 Source HGNC Symbol Acc HGNC 7999
F09	SBH0148171	ENST00000303212.2	NRTN	ENSG00000171119	neurturin Source HGNC Symbol Acc HGNC 8007
F10	SBH0012802	ENST00000543548.1	NTF3	ENSG00000185652	neurotrophin 3 Source HGNC Symbol Acc HGNC 8023
F11	SBH0048137	ENST00000565123.5	OSGIN1	ENSG00000140961	oxidative stress induced growth inhibitor 1 Source HGNC Symbol Acc HGNC30093
F12	SBH0518114	ENST00000506880.5	PDGFC	ENSG00000145431	platelet derived growth factor C Source HGNC Symbol Acc HGNC 8801
G01	SBH1220303	ENST00000238607.10	PGF	ENSG00000119630	placental growth factor Source HGNC Symbol Acc HGNC 8893
G02	SBH0231621	ENST00000597721.1	PSPN	ENSG00000125650	persephin Source HGNC Symbol Acc HGNC 9579
G03	SBH0080420	ENST00000348225.6	PTN	ENSG00000105894	pleiotrophin Source HGNC Symbol Acc HGNC 9630
G04	SBH0646520	ENST00000453443.5	SLCO1A2	ENSG00000084453	solute carrier organic anion transporter family member 1A2 Source HGNC Symbol Acc HGNC 10956
G05	SBH0180162	ENST00000237623.11	SPP1	ENSG00000118785	secreted phosphoprotein 1 Source HGNC Symbol Acc HGNC 11255
G06	SBH0577809	ENST00000471721.1	TDGF1	ENSG00000241186	teratocarcinoma-derived growth factor 1 Source HGNC Symbol Acc HGNC 11701
G07	SBH1220443	ENST00000598758.5	TGFB1	ENSG00000105329	transforming growth factor beta 1 Source NCBI gene Acc 7040
G08	SBH0321723	ENST00000647395.1	THPO	ENSG00000090534	thrombopoietin Source HGNC Symbol Acc HGNC 11795
G09	SBH0332854	ENST00000587758.5	TNNT1	ENSG00000105048	troponin T1, slow skeletal type Source HGNC Symbol Acc HGNC 11948
G10	SBH1220500	ENST00000395681.6	TYMP	ENSG00000025708	thymidine phosphorylase Source HGNC Symbol Acc HGNC 3148
G11	SBH0420322	ENST00000425836.6	VEGFA	ENSG00000112715	vascular endothelial growth factor A Source HGNC Symbol Acc HGNC 12680
G12	SBH1220517	ENST00000618562.2	VEGFC	ENSG00000150630	vascular endothelial growth factor C Source HGNC Symbol Acc HGNC 12682
H01	SBH1220543	ENST00000646664.1	ACTB	ENSG00000075624	actin beta Source HGNC Symbol Acc HGNC 132
H02	SBH1220550	ENST00000558401.6	B2M	ENSG00000166710	beta-2-microglobulin Source HGNC Symbol Acc HGNC 914
H03	SBH1220545	ENST00000396861.5	GAPDH	ENSG00000111640	glyceraldehyde-3-phosphate dehydrogenase Source HGNC Symbol Acc HGNC 4141
H04	SBH1220546	ENST00000298556.8	HPRT1	ENSG00000165704	hypoxanthine phosphoribosyltransferase 1 Source HGNC Symbol Acc HGNC 5157
H05	SBH1220553	ENST00000546989.5	RPLP0	ENSG00000089157	ribosomal protein lateral stalk subunit P0 Source HGNC Symbol Acc HGNC 10371
H06	SBH1218553	Sybr_HGDC	HGDC	Sybr_HGDC	Human Genomic DNA Contamination
H07	SBH1218551	Sybr_QIC	QIC	Sybr_QIC	QuantiNova Internal Control
H08	SBH1218551	Sybr_QIC	QIC	Sybr_QIC	QuantiNova Internal Control
H09	SBH1218551	Sybr_QIC	QIC	Sybr_QIC	QuantiNova Internal Control
H10	SBH1218550	Sybr_PPC	PPC	Sybr_PPC	Positive PCR Control
H11	SBH1218550	Sybr_PPC	PPC	Sybr_PPC	Positive PCR Control
H12	SBH1218550	Sybr_PPC	PPC	Sybr_PPC	Positive PCR Control

### Statistical analysis

Results are presented as mean ± SEM unless otherwise stated. Significance comparison was analysed using one-way ANOVA. Data were processed and analysed in GraphPad Prism. *P* < 0.05 was considered as statistically significant difference (*); *P* < 0.01 (**); *P* < 0.001 (***).

## Results

### Effects of single AFB_1_ and single FB_1_ treatments on cell viability

**Figure 1.**
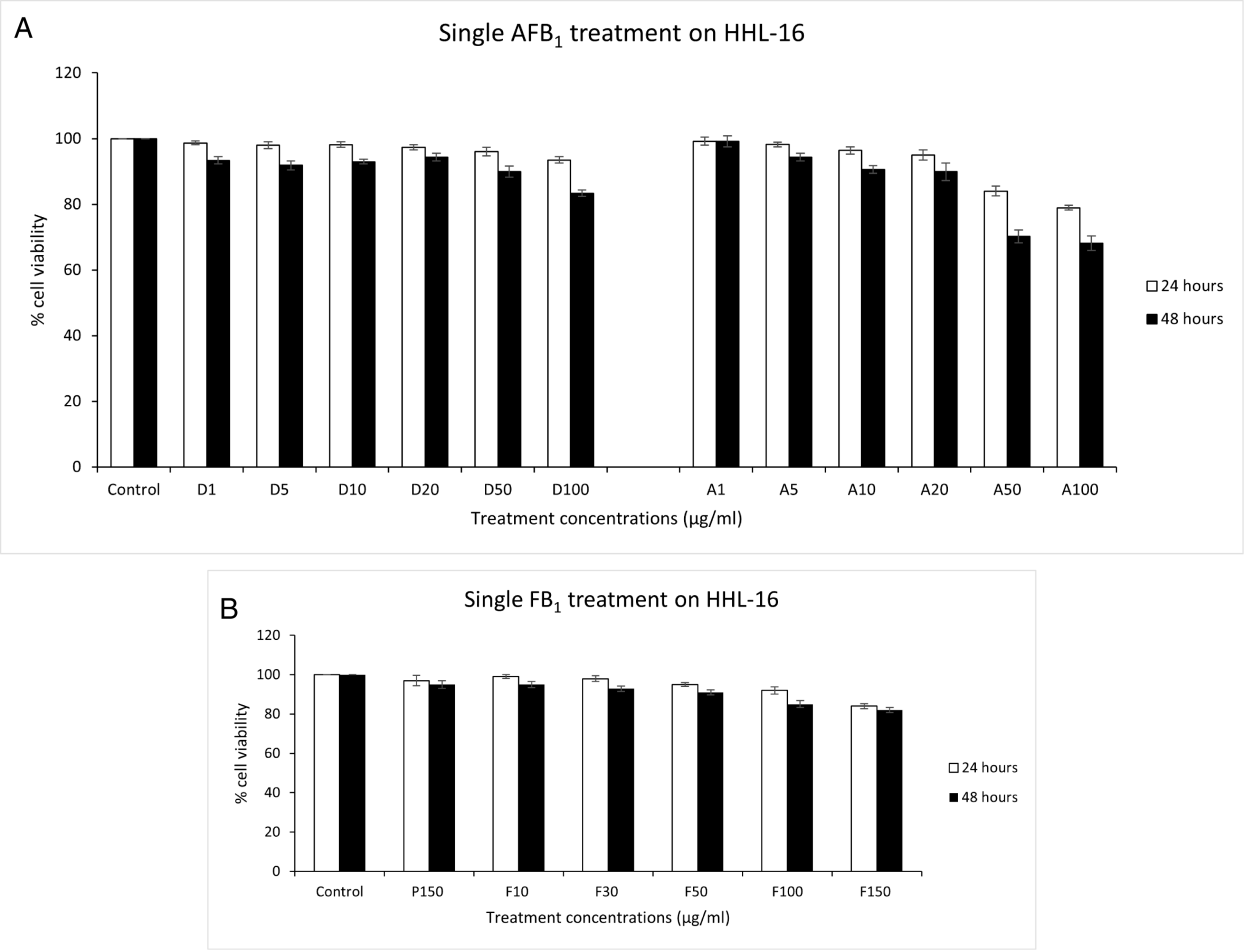
Single AFB_1_ (A) or FB_1_ (B) treatment on HHL-16 cells for 24 and 48 h. Control: untreated cells; D: DMSO treatments; A: AFB_1_ treatments; P: PBS treatment; F: FB_1_ treatment. DMSO and PBS treatments were normalized against the control group. AFB_1_ treatments were normalized against corresponding DMSO treatments (i.e. A1 against D1), and FB_1_ treatments were normalized against the PBS treatment. With the increasing of either AFB_1_/FB_1_ concentrations or treated time, the cell viability was decreased, so there were dose-dependent and time-dependent cytotoxicity of both AFB_1_ and FB_1_ in HHL-16 cells. Data are presented as mean ± SEM, and the results were repeated three times.

To investigate the toxicity pattern of AFB_1_ and FB_1_ on HHL-16 cells, cell viability was determined by MTT assay in HHL-16 cells exposed to single AFB_1_ (1, 5, 10, 20, 50, and 100 µg/ml) and FB_1_ (10, 30, 50, 100, and 150 µg/ml) for 24 and 48 h. Either with the increasing of aflatoxin concentration from 1 to 100 µg/ml or treatment time from 24 to 48 h, the cell viability was decreased, demonstrating a dose-dependent and time-dependent toxicity of aflatoxin in HHL-16 cells (Fig. 1A). With the toxicity pattern of FB_1_, decreased cell viability with increasing concentration and time was also observed, so there is also a dose-dependent and time-dependent manner of fumonisin toxicity in HHL-16 cells (Fig. 1B).

### Effects of combined AFB_1_ and FB_1_ treatments on cell viability

**Figure 2.**
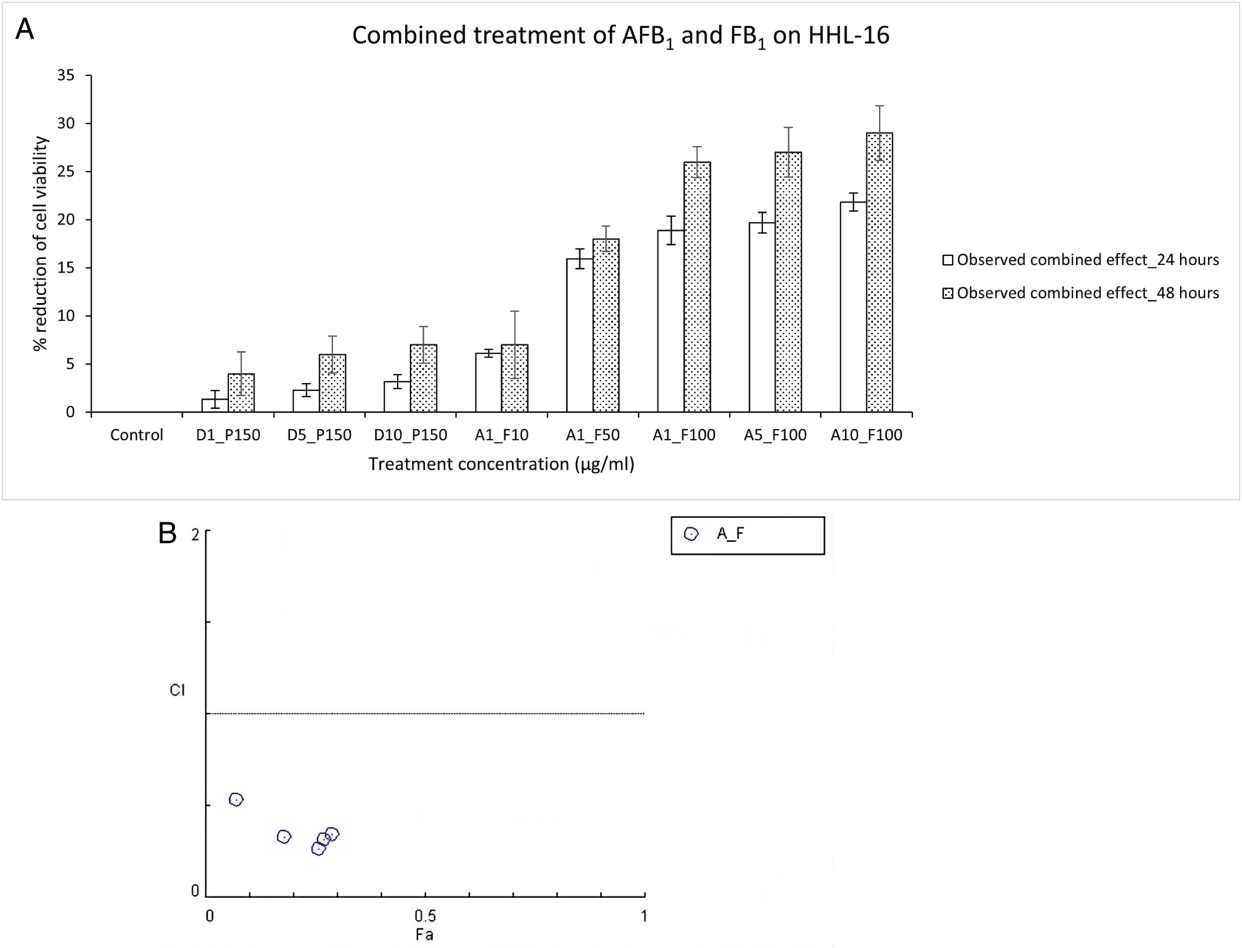
(A) Combined treatment of AFB_1_ and FB_1_ on HHL-16 cells for 24 and 48 h. Control: untreated cells; D: DMSO treatment; P: PBS treatment; A: AFB_1_ treatment; F: FB_1_ treatment; PBS and DMSO treatment were normalized against control groups. Combined treatments were normalized against corresponding combined treatment of DMSO and PBS. Expected additive effect: the sum of the toxic effect of single AFB_1_ and FB_1_. Combined AFB_1_ and FB_1_ treatment has a synergistic effect on HHL-16 cells 24 and 48 h post the treatment, it is both dose-dependent and time-dependent manner. Data was presented as mean ± SE, and the results were repeated three times. (B) Combinatory effects of aflatoxin and fumonisin evaluated by the combination index (CI) theorem. Fa: fraction affected (Fa = 1 − inhibition of cell viability%)/100. CI < 1, =1, >1 represent for synergistic, additive, and antagonistic effects, respectively. A synergistic effect was observed in the combination of AFB_1_ and FB_1_ in HHL-16 cells.

To determine the combined effects of AFB_1_ and FB_1_, different combinations of AFB_1_ and FB_1_ (A1_F10, A1_F50, A1_F100, A5_100, and A10_F100 µg/ml) were used to treat HHL-16 cells for 24 and 48 h. Cell viability was assessed by the MTT assay. The result shows that either a fixed concentration of AFB_1_ with an increased concentration of FB_1_, or a fixed concentration of FB_1_ with an increased concentration of AFB_1_, both caused a greater reduction of cell viability (Fig. 2A). In addition, the observed combined effects of AFB_1_ and FB_1_ caused a greater reduction of cell viability after 48 h of treatment compared to the cell viability after 24 h of treatment (Fig. 2A). Therefore, with the increasing combined concentrations or treatment time, a greater reduction of cell viability was caused, which suggests the combined effect of AFB_1_ and FB_1_ toxicity was dose-dependent and time-dependent in HHL-16 cells. CI values for combined AFB_1_ and FB_1_ treatments in HHL-16 cells were determined and summarized (Tables 3 and 4). As the result shows (Fig. 2B) the CI values of the five non-constant combinations of AFB_1_-FB_1_ are all lower than 1, confirming a synergistic interaction of the combination.

**Table 3.** Combination index values calculated by CompuSyn in the five combined AFB_1_ and FB_1_ treatments on HHL-16 cells for 24 h.

Mycotoxin	Combination ratio	24 h treatment	
		CI	DRI
AFB_1__1 µg/ml	1:10	0.21	16.6
FB_1__10 µg/ml			6.5
AFB_1__1 µg/ml	1:50	0.29	72.1
FB_1__50 µg/ml			3.6
AFB_1__1 µg/ml	1:100	0.47	95.4
FB_1__100 µg/ml			2.2
AFB_1__5 µg/ml	1:20	0.48	20.8
FB_1__100 µg/ml			2.3
AFB_1__10 µg/ml	1:10	0.47	12.2
FB_1__100 µg/ml			2.6

**Table 4.** Combination index calculated by CompuSyn in the five combined AFB_1_ and FB_1_ treatments on HHL-16 cells for 48 h.

Mycotoxin	Combination ratio	48 h treatment	
		CI	DRI
AFB_1__1 µg/ml	1:10	0.53	8.7
FB_1__10 µg/ml			2.4
AFB_1__1 µg/ml	1:50	0.33	31.2
FB_1__50 µg/ml			3.4
AFB_1__1 µg/ml	1:100	0.27	54.7
FB_1__100 µg/ml			4.0
AFB_1__5 µg/ml	1:20	0.31	11.6
FB_1__100 µg/ml			4.4
AFB_1__10 µg/ml	1:10	0.34	6.6
FB_1__100 µg/ml			5.3

### mRNA expression of cytochrome P450 (CYP 450) genes

**Figure 3.**
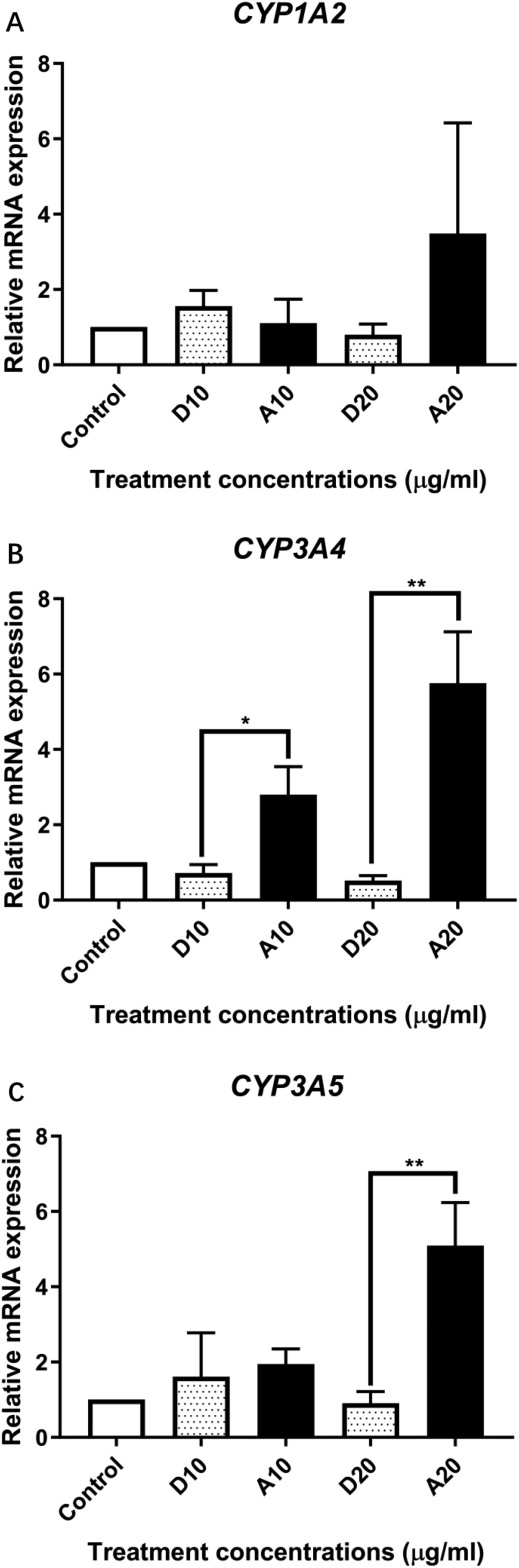
Gene expression of cytochrome P450 (CYP) enzymes in HHL-16 cells exposed to aflatoxin for 24 h. Control: untreated cells; D: DMSO treatment; A: aflatoxin treatment. *ACTB*: Beta-actine, a reference gene used for the normalization to *CYP1A2* (A), *CYP3A4* (B) and *CYP3A5* (C). A significant increase of *CYP3A4* and *CYP3A5* were observed in HHL-16 cells after aflatoxin treatment for 24 h. Although there is a tendency to be higher expression of *CYP1A2*, it did not reach significant level.

In humans, aflatoxin is mainly metabolized by cytochrome P450 enzymes to produce toxic metabolites, so to investigate whether AFB_1_ is metabolized in the cell line, the gene expression of key CYP genes including *CYP1A2*, *3A4*, and *3A5* was determined in HHL-16 cells after AFB_1_ treatments. The qPCR result (Fig. 3) shows that the maximal level of gene expression fold change was observed in *CYP3A4* gene. Significantly increased expression of *CYP3A4* (5.76 folds, *P* = 0.0027) and *3A5* (5.09 folds, *P* = 0.0037) was observed in the cells after 20 µg/ml AFB_1_ treatment compared to the control groups. The changes observed in gene expression of *CYP1A2* did not reach statistical significance.

### Differential mRNA expression of growth factors pathway in HHL-16 cells after individual AFB_1_ or FB_1_ treatment

**Figure 4.**
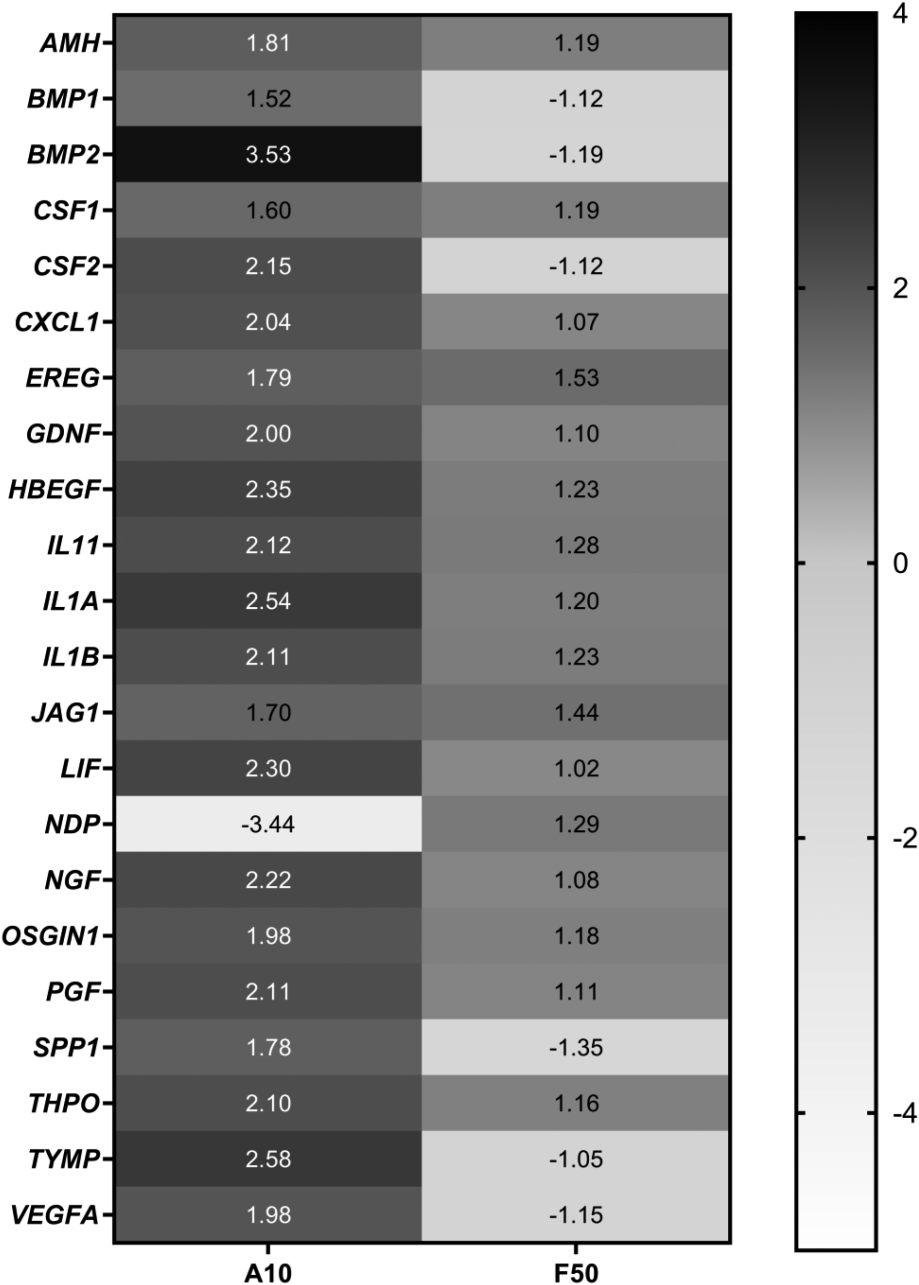
Heat map of gene expression fold change of several selected genes in growth factors pathway in HHL-16 cells exposed to single AFB_1_ (10 µg/ml) or single FB_1_ (50 µg/ml) for 24 h. A: AFB_1_ treatment; F: FB_1_ treatment. The colours represent the up-regulated (black) and down-regulated (white) genes compared to the control group, respectively.

To validate the hypothesis that gene expression of human growth factors could be altered by AFB_1_ and/or FB_1_ treatments in the cells, human growth factors pathway-focussed gene expression profiling was analysed with the QuantiNova^®^ LNA^®^ PCR Panels in HHL-16 cells after individual AFB_1_ or FB_1_ treatment. The differentially expressed genes were identified by fold changes greater than 2-fold. HHL-16 cells treated with 10 µg/ml AFB_1_ showed significantly differential gene expression pattern compared to the control, while there was no significant gene expression change in the cells after 50 µg/ml FB_1_ treatment for 24 h (Fig. 4). Fifteen genes (e.g. *BMP2*, *CSF2*, *IL1A*, and *TYMP*) were identified to be up-regulated as fold change of the gene expression was greater than 2, with the highest upregulation of *BMP2* observed after a single AFB_1_ treatment. Additionally, the most significant decrease in *NDP* gene expression was found in the cells after AFB_1_ treatment for 24 h. Based on these results, *BMP2* and *NDP* genes were further analysed using RT-qPCR.

### Changes in mRNA expression of candidate genes in HHL-16 cells after single AFB_1_ or FB_1_ treatment

**Figure 5.**
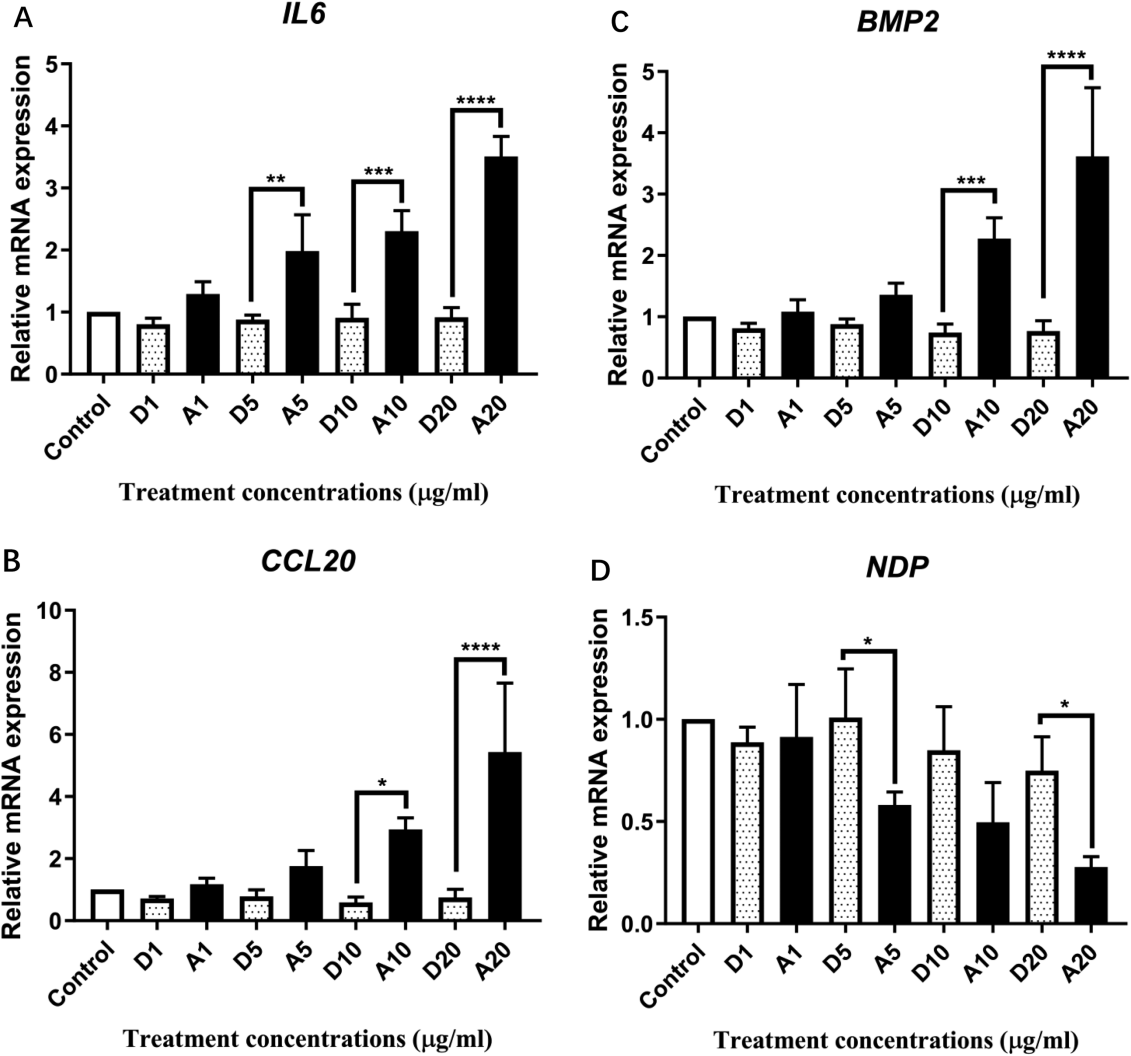
Differential gene expression of candidate genes, *IL6* (A), *CCL20* (B), *BMP2* (C), and *NDP* (D), involved in immune and growth factors pathways in HHL-16 cells exposed to AFB_1_ for 24 h. Control: untreated cells. D: DMSO treatments. A: AFB_1_ treatments. *GAPDH* and *ACTB*, two reference genes were used to normalize the gene expression of *IL6*, *CCL20* and *NDP*. Dose-dependent increasing of *IL6*, *CCL20* and *BMP2*, and dose-dependent decreasing of *NDP* were observed.

Previous data in our lab found that *IL6* and *CCL20* were significantly differentially expressed in HHL-16 cells after a single AFB_1_ treatment [44,45]. To confirm the findings of our pathway-focussed gene expression analysis from both human growth factors and immune-related pathways, the differentially expressed genes were further assessed by RT-qPCR. As shown in Fig. 5, the results are consistent with the results of the pathway screening. mRNA expression of *IL6*, *CCL20*, and *BMP2* were significantly increased and *NDP* was significantly decreased after AFB_1_ treatments, which were dose-dependent effects. However, there was no significant difference in mRNA expression level of the four candidate genes (*IL6*, *CCL20*, *BMP2*, and *NDP*) in the cells after a single FB_1_ treatment for 24 h, which is also consistent with the pathway screening results (Supplementary Fig. 1).

### Changes in mRNA expression of candidate genes in HHL-16 cells after combined AFB_1_ and FB_1_ treatment

**Figure 6.**
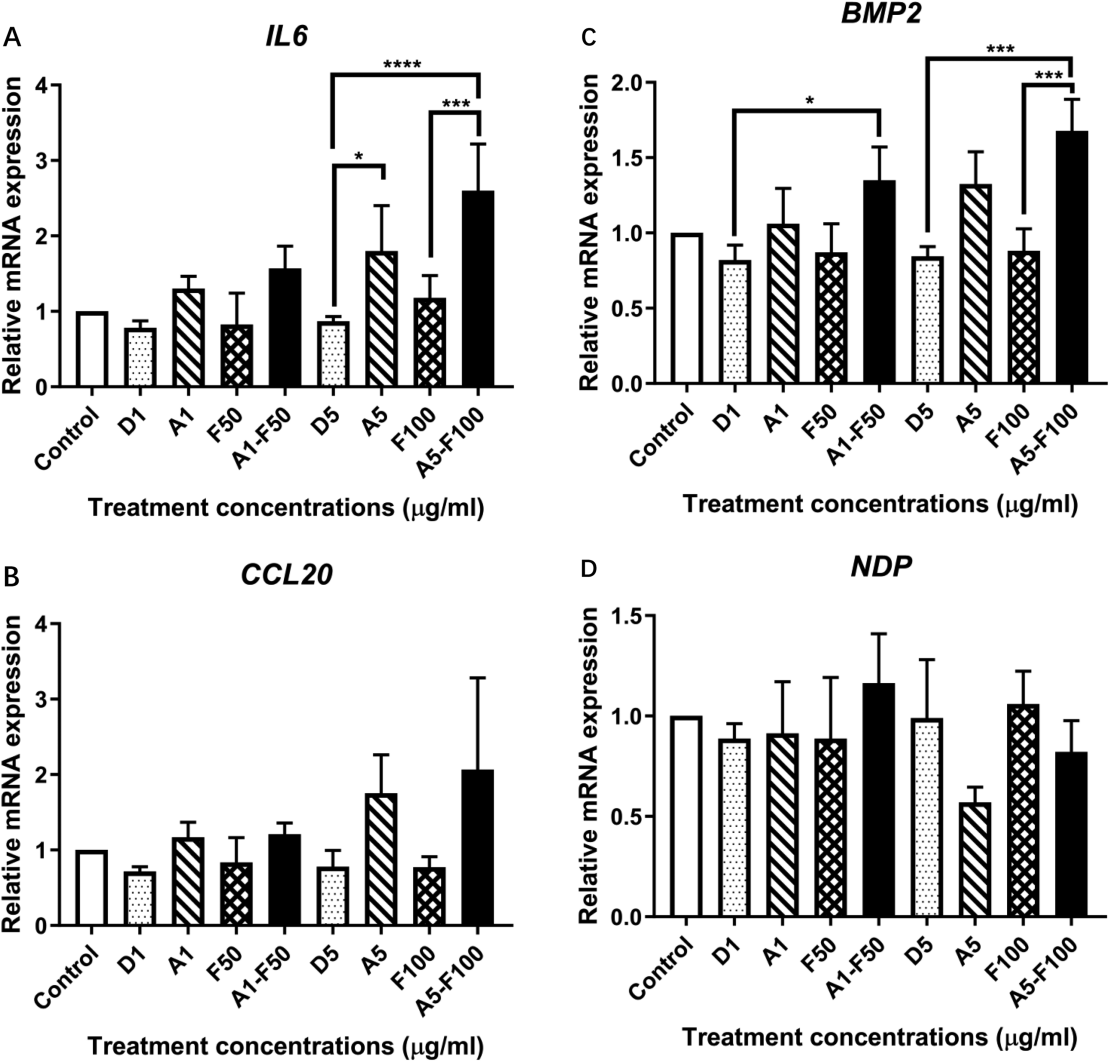
Differential gene expression of candidate genes, *IL6* (A), *CCL20* (B), *BMP2* (C), and *NDP* (D), involved in immune and growth factors pathways in HHL-16 cells exposed to both AFB_1_ and FB_1_ for 24 h. Control: untreated cells. D: DMSO treatments. A: AFB_1_ treatments. F: FB_1_ treatments. *GAPDH* and *ACTB*, two reference genes were used to normalize the gene expression of *IL6*, *CCL20*, *BMP2* and *NDP*. Dots, diagonal lines, crosshatch, and black columns represent the control, single AFB_1_, single FB_1_, and combined AFB_1_ and FB_1_ treatment, respectively. *IL6* and BMP2 expression levels were significantly higher after the combined treatment of 5 µg/ml AFB_1_ and 100 µg/ml FB_1_ than 100 µg/ml FB_1_ treatment alone and had a tendency to be higher compared to the individual 5 µg/ml AFB_1_ treatment.

*IL6* gene expression level was significantly higher in the combined treatment of 5 µg/ml AFB_1_ and 100 µg/ml FB_1_ than the individual FB_1_ treatment (100 µg/ml) and had a tendency to be higher than 5 µg/ml AFB_1_ treatment alone (Fig. 6). Although the gene expression fold change of *BMP2* in combined treatment of 5 μg/ml AFB_1_ and 100 μg/ml FB_1_ did not reach 2-fold, it was found that the mRNA level of *BMP2* was significantly higher in the combined treatment than individual 100 μg/ml FB_1_ treatment (Fig. 6). However, there was no significant difference in mRNA expression observed in *CCL20* and *NDP* genes after the combined treatments of AFB_1_ and FB_1_ compared to the individual treatments.

## Discussion

The main findings from this study were: (i) combined AFB_1_ and FB_1_ treatments showed synergistic toxicity in HHL-16 cells. (ii) Combined treatment of HHL-16 cells with AFB_1_ and FB_1_ led to higher mRNA expression of *IL6* and *BMP2* genes compared to individual AFB_1_ or FB_1_ treatments. To date, most mycotoxin liver cell toxicity studies have used HepG2 cells, a human liver carcinoma cell line. Here, we have used the non-tumorigenic cell line, HHL-16 cells, to reduce the influence of cancer-related changes on gene expression, as HHL-16 cells retain primary hepatocyte characteristics, which express hepatocyte-specific makers and CYP450 enzymes necessary for aflatoxin bio-activation [39]. Comparison with previous results in HepG2 cells shows that the HHL-16 cells are less sensitive to both AFB_1_ and FB_1_ than HepG2 cells [46–49].

Combined effects of AFB_1_ and FB_1_ on cytotoxicity have been found to be either additive or synergistic in various animal studies; different responses might be associated with the various testing methods or binary concentrations used, and/or species differences [50,51]. In broilers, AFB_1_ and FB_1_ in combination had additive toxic effects on liver structure, body weight, and immunological response [52], whereas a synergistic effect of AFB_1_ and FB_1_ on liver damage and reduced weight gain was seen in male F344 rats [34]. In the present study, our results found that there was a synergistic cytotoxicity of AFB_1_ and FB_1_ in HHL-16 cells, which is in line with the synergistic interactions of AFB_1_ and FB_1_ described in previous animals and cell line studies [35,49,53]. The combined toxicity of AFB_1_ and FB_1_ shown here highlights the potential hazard from combined contamination of crops with these two mycotoxins.

Gene expression of *IL6*, *CCL20*, and *BMP2* was significantly increased in HHL-16 cells after AFB_1_ treatments for 24 h, while *NDP* gene expression was significantly decreased. Increased *IL6* gene expression has previously been reported in broilers and pigs fed with AFB_1_-contaminated diet [54,55]. IL6 is an important pro-inflammatory cytokine and plays key roles in the process of hepatic inflammation. Increased production of IL6 was correlated with liver lesions in rats fed with an intermittent dosing regimen of AFB_1_ [56]. Therefore, the observation of increased mRNA expression of *IL6* in HHL-16 cells may support the hypothesis that the inflammatory response is associated with the hepatoxicity of AFB_1_. Increased expression of *CCL20*, which we report herein HHL-16 cells, has been seen in chickens administrated with AFB_1_ [57]. CCL20, a chemokine, can be induced by the combination of IL6 and its soluble receptor (sIL-6R) [58]. In astrocytes, it was found that IL6 combined with sIL-6R significantly increased expression of CCL20 through the activation of STAT3 and binding of phosphorylated STAT3 to the promoter of CCL20 [58]. Elevated CCL20 production and its receptor CCR6 have been related to the progression of a variety of human cancers, including liver cancer, colorectal cancer, breast cancer, and kidney cancer, and also indirectly modulates the function of immune cells in response to inflammatory diseases [59,60]. Therefore, the increased expression of *IL6* and *CCL20* in response to AFB_1_ may play a role in AFB_1_-related inflammatory diseases and cancers.

Our study is the first to find aberrant expression of *BMP2* and *NDP* in cells after AFB_1_ treatments. Increased gene expression of *BMP2* has been reported in human osteoblasts treated with cadmium and in patients with non-alcoholic fatty liver disease (NAFLD) [61,62]. Recently, evidence indicates that BMP2 plays a dynamic role in liver physiology. Transient decreased expression of BMP2 after partial hepatectomy was found during the process of liver regeneration in rats, while upregulation of BMP2 was observed during carbon tetrachloride (CCl_4_)-induced fibrosis in rats, in mice after chronic alcohol exposure, and in NAFLD patients [62–64]. Therefore, the increased mRNA expression of *BMP2* we observed after AFB_1_ treatments of HHL-16 cells might indicate a novel mechanism in aflatoxin-induced chronic liver injury. The other differentially expressed gene, *NDP*, is a potent upstream mediator of Notch signalling, which is a highly conserved pathway crucial in development and implicated in malignant transformation [65,66]. Suppressed *NDP* gene expression may affect downstream growth and development and contribute to disease onset. The aberrant gene expression changes that we have observed in HHL-16 cells following AFB_1_ may reflect the impact of aflatoxin on liver injury, immune modulation, growth failure, and cancers could be at least in part be due to disruption of cytokines, chemokine, and growth factors.

We did not find any significant effects of individual FB_1_ on gene expression of human growth factors pathway and the two immune-related genes (*IL6* and *CCL20*) in HHL-16 cells. No significant change of gene expression was found in our study might be because of the species differences and variations [67].

Despite there being no significant changes in gene expression induced by FB_1_ on its own, the combined treatment induced higher mRNA levels of *IL6* and *BMP2* in HHL-16 cells compared to the individual treatments, suggesting a synergistic effect of AFB_1_ and FB_1_ on transcription of *IL6* and *BMP2*. Synergistic induction of IL6 could be a reason for more cell apoptosis caused by the mixture of mycotoxins, which will contribute to a greater inflammatory response [68]. IL6 has been shown to enforce proliferation and anti-apoptotic effects in tumour cells to promote tumour progression [69]. The evidence of elevated *IL6* expression in our study might shed light on the mechanism of higher incidence of cancers reported in animals and humans exposed to both AFB_1_ and FB_1_. BMP2 is a growth factor and plays an important role in bone formation, but recent studies found that BMP2 might also play a role in carcinoma progression. Higher BMP2 expression was found in the liver of patients with human hepatocellular carcinoma than that of normal liver tissues, and exogenous BMP2 injected into mice enhanced liver cancer growth through the activation of myeloid-derived suppressor cells to secret more IL6, which could promote cell proliferation of liver cancer cells [70]. Uncontrolled cell proliferation in healthy cells is a hallmark of the transformation to cancer cells [71]. In this study, although *BMP2* expression was not induced by FB_1_, the mixture of AFB_1_ and FB_1_ induced higher *BMP2* gene expression compared to FB_1_ treatment alone, indicating FB_1_ might have the potential to increase the carcinogenicity of AFB_1_. Therefore, we deduced that combination of AFB_1_ and FB_1_ would cause more inflammatory response and increase cell proliferation to enhance the carcinogenicity through the activation of IL6 and BMP2 signalling. However, analysis *in vitro* may not represent the full biological effects of IL6 and BMP2 *in vivo*, as any interaction between them to promote cancer will be more complex *in vivo*. Further studies *in vivo* to replicate the results in this study are warranted.

In summary, the present results indicate that AFB_1_ and FB_1_, either alone or in combination, had dose-dependent and time-dependent toxicity in HHL-16 cells. Combination of AFB_1_ and FB_1_ showed a synergistic toxicity in the cells. AFB_1_ increased transcription of *IL6*, *CCL20*, *BMP2* and decreased *NDP* gene expression level. Combined treatment with AFB_1_ and FB_1_ showed a synergistic induction of mRNA expression levels of *IL6* and *BMP2*, suggesting that the presence of FB_1_ may increase the cytotoxicity of AFB_1_ through increasing the inflammatory response and disrupting the cell growth balance. More attention needs to be paid to the co-occurrence of aflatoxin and fumonisin in natural diets, and further studies *in vivo* should be conducted to confirm the findings obtained in this study.

## Supplementary data

Supplementary data is available at *Mutagenesis* online.

geae005_suppl_Supplementary_File

## Data Availability

The data will be shared upon request to the corresponding author.
