# Supplementary figures and images for: Effects of aflatoxin and fumonisin on gene expression of growth factors and inflammation-related genes in a human hepatocyte cell line
Source: Mutagenesis. 2024 Mar 12;39(3):181–95. doi: 10.1093/mutage/geae005 (PMC11040159; doi:10.1093/mutage/geae005)

Supplementary Figure

A

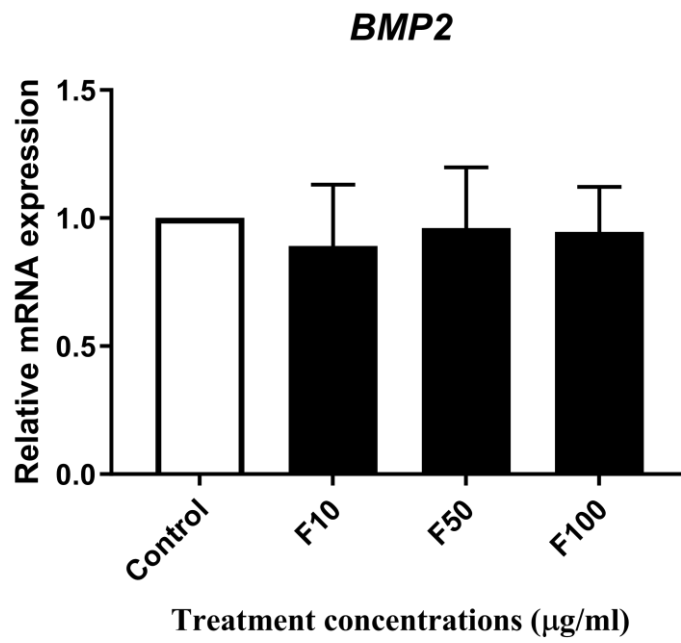

B

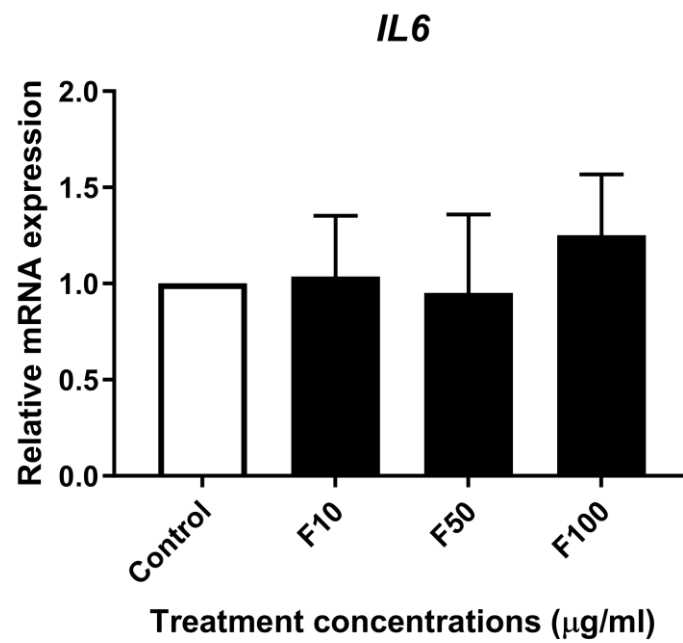

C

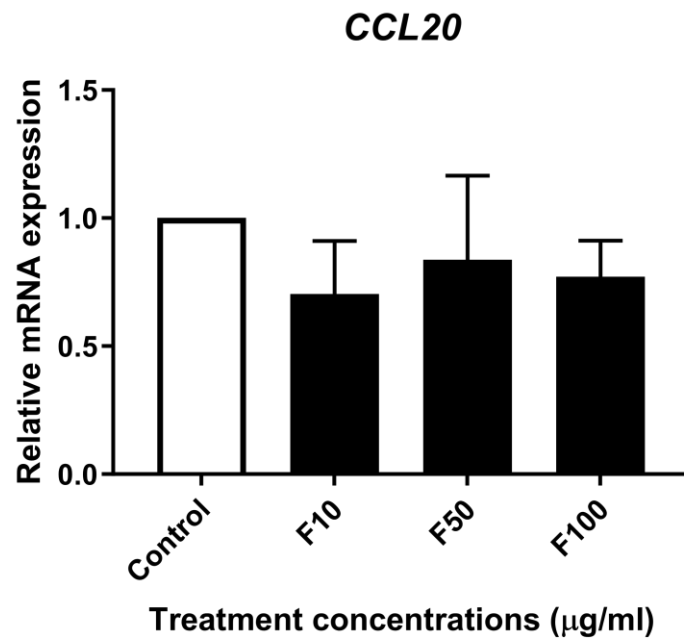

D

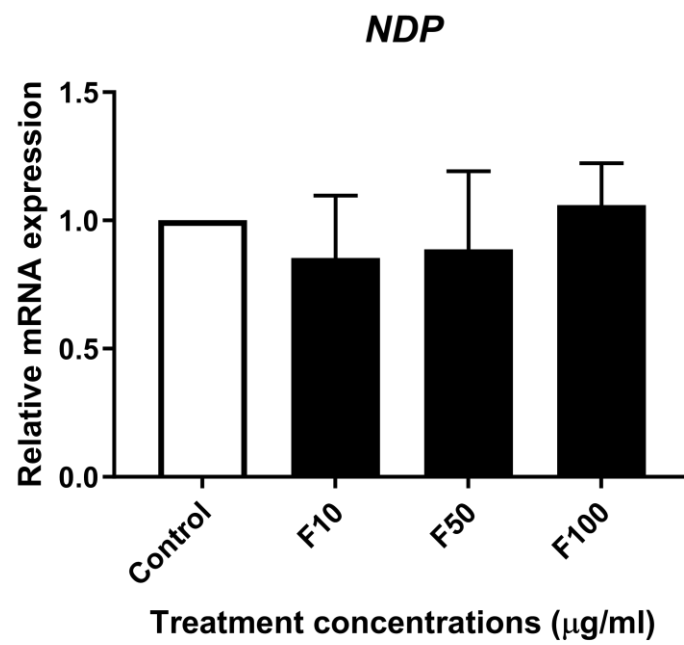

Supplement: geae005_suppl_Supplementary_File [file geae005_suppl_supplementary_file.pdf]
